# Spinal Stroke following Kidney Transplant

**DOI:** 10.1155/2022/2058600

**Published:** 2022-05-21

**Authors:** Jayanthan B. Subramanian, Farjad Siddiqui, Pranit N. Chotai, Yazan Al-Adwan, Amer Rajab, Kenneth Washburn, Austin D. Schenk, Ashley J. Limkemann, Michael Luttrull, Musab Al-Ebrahim, Ginny Bumgardner, Navdeep Singh

**Affiliations:** ^1^Division of Transplantation, Department of Surgery, The Ohio State University Wexner Medical Center, USA; ^2^Department of Radiology, The Ohio State University Wexner Medical Center, USA

## Abstract

Complications are a part of surgery. Spinal infarctions are a dreaded complication of aortic surgery. We present a patient who developed a spinal infarct after a kidney transplant. We were unable to find a causative factor in our search for etiology. In our review of the literature, we were unable to find a similar report. We present this case report to highlight a rare complication of kidney transplantation and to reinforce that patients requiring kidney transplant are complex patients with multiple comorbidities that can cause a multitude of complications in the periop period.

## 1. Introduction

Kidney transplants are the most common solid organ transplants performed across the United States with over 23000 transplants done in 2021 as per OPTN (Organ Procurement and Transplantation Network) data. Various common and uncommon complications have been described in kidney transplant recipients [1]. We present a rare complication in a kidney transplant recipient who developed an infarct of his spinal cord following his kidney transplant leading to profound morbidity in the patient.

## 2. Case Report

Our patient is a 32 y/o male with end-stage renal disease secondary to DM type 2. Past medical history was significant for congestive heart failure (CHF), diabetic neuropathy with foot ulcer, and obstructive sleep apnea (OSA). He was on hemodialysis (HD) since 10/2017. He was evaluated by our multidisciplinary team and approved for a kidney transplant. A suitable donor became available, and he underwent kidney transplant in March 2021.

The surgery was performed using the standard technique; the renal artery was anastomosed to the external iliac artery in an end to side fashion. The renal vein was anastomosed to the external iliac vein in an end to side fashion. The ureter was anastomosed to the bladder and imbricated to form a nonrefluxing anastomosis.

The surgery was uneventful. Intraoperatively, the patient remained hemodynamically stable with no episodes of hypotension; the patient had an average MAP (mean arterial pressure) of around 75 mmHg. Reperfusion of the kidney perfused well.

On postoperative day 1, the patient complained of weakness of both his legs and loss of sensation. On examination, the patient had normal muscle tone, power of 0/5 throughout his lower limbs, and absent lower limb reflexes.

Light, touch, temperature, and pin-prick sensations were absent below the midthigh bilaterally while vibration was reduced throughout bilaterally.

A CT AP was done, which was normal.

**Figure 1:**
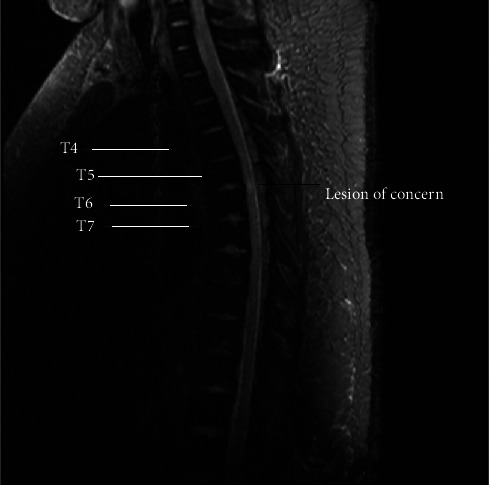
T2 MRI STIR.

**Figure 2:**
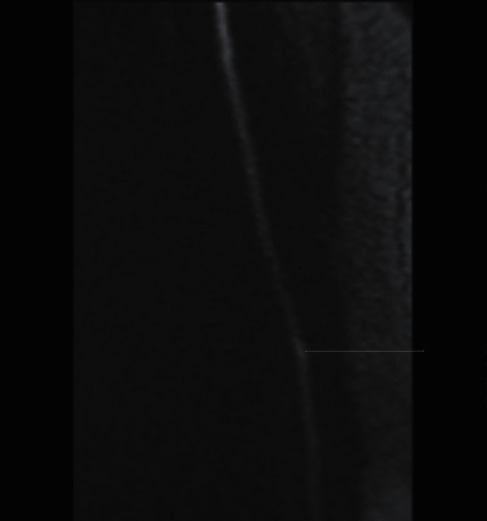
MRI diffusion-weighted imaging.

An MRI of the brain and the spine was done which showed a small basal ganglia infarct and an infarct of the spinal cord at the level of T5-6 (Figures 1 and 2)

Neurology and neurosurgery were consulted; based on their recommendation, the patient was planned to be managed conservatively and maintain his MAP > 85 mmHg.

**Figure 3:**
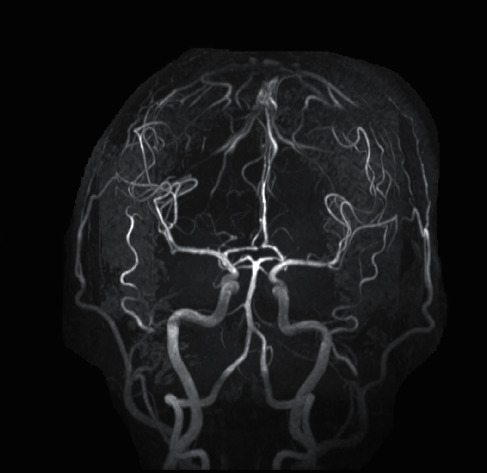
MR angiography of cerebrospinal vessels.

A MR angiogram of the cerebrospinal vessels was performed which did not show any significant stenosis Figure 3.

An echocardiogram was done which was significant for left ventricular hypertrophy. A bubble study done at the time did not demonstrate any shunts.

**Figure 4:**
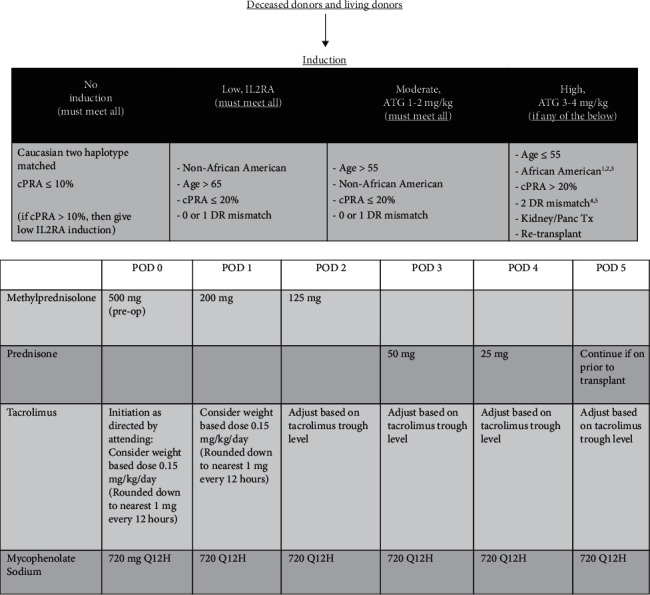
Induction protocol.

The patient received induction immunosuppression with ATG and prednisone taper as per our institution protocol and maintenance immunosuppression with mycophenolate and tacrolimus (Figure 4).

He had delayed graft function and required dialysis twice, and kidney function stabilized without the need for dialysis by the time of discharge.

The patient was discharged to a rehab facility for spinal cord rehab. At the time of discharge, he had a power of 1/5. He needed intermittent straight catheterizations to drain his bladder and required suppositories every few days to help with his defecation.

He was readmitted twice, for a UTI the first time. He was evaluated for a diabetic foot ulcer with concerns for osteomyelitis in this admission; a foot MRI was done which showed osteomyelitis of the fourth and fifth metatarsal heads. He was not willing for debridement and resection of the infected bone and instead preferred prolonged IV antibiotics to treat this.

He was admitted for the second time with worsening sacral decubitus ulcers, and a diverting loop colostomy was done to aid with healing. His nonhealing diabetic foot ulcer was again reassessed. He underwent wound debridement and ray amputation of the fourth and fifth toes during this admission.

He continues to be followed up in clinic. At the time of submission, he continues to live in a rehab facility; his kidney function is at his posttransplant baseline.

## 3. Discussion

This is a very rare and devastating complication occurring after any surgery. As the posttransplant workup was inconclusive, we were not able to pinpoint the cause for this complication which most probably seem to be a result of an embolism. In our search of the literature, we were unable to find reports of a spinal infarction complicating a kidney transplant.

Many complications are known to occur in this population. A meta-analysis done by Mohammadi et al. in 2019 [1] found that peripheral neuropathy was the most common neurological complication followed by tremors and cerebrovascular accidents [2]. Infectious complications were seen to occur in tropical areas amongst populations with lower socioeconomic backgrounds [3].

Immunosuppressive drug toxicity is directly related to certain disorders with calcineurin inhibitors causing tremors and paresthesia [2, 4].

Cerebrovascular accident occurs in around 8% of kidney transplant patients with diabetes, hypertension, and the accelerated atherosclerosis seen in many transplant candidates being a contributing factor [5].

Spinal infarction has been commonly associated with aortic aneurysm surgery, other aortic surgeries, spinal surgeries, and thoracic and cardiac surgeries which involved manipulation/mobilization of the aorta [5, 6], but our patient did not have any of this.

Other procedures which are commonly complicated by spinal cord infarctions are epidural injections, nerve blocks, and embolizations [7]. There is a report of spinal cord infarction following repair of bilateral iliac aneurysm repair [7].

Intraprocedural hypotension and thromboembolic phenomenon are the most common pathologic mechanisms behind spinal infarctions [8–10]. Our patient did not have intraoperative hypotension. In view of his long-standing T2DM and its associated microvascular complication such has nephropathy, neuropathy, and retinopathy, it was thought that he may have had small vessel disease which contributed to his spinal infarction and the possibility of likely embolization cannot be ruled out.
